# A genome-wide association study of chemotherapy-induced alopecia in breast cancer patients

**DOI:** 10.1186/bcr3475

**Published:** 2013-09-11

**Authors:** Suyoun Chung, Siew-Kee Low, Hitoshi Zembutsu, Atsushi Takahashi, Michiaki Kubo, Mitsunori Sasa, Yusuke Nakamura

**Affiliations:** 1 Department of Medicine, The University of Chicago, 5801 South Ellis Avenue, Chicago, IL 60637, USA; 2 Department of Surgery, The University of Chicago, A27 S Maryland Avenue, Chicago, IL 60637, USA; 3 Laboratory for Statistical Analysis, Center for Genomic Medicine, 1-7-22 Suehiro-cho, Tsurumi-ku, Yokohama, Kanagawa 230–0045, Japan; 4 Laboratory for Genotyping Development, Center of Genomic Medicine, 1-7-22 Suehiro-cho, Tsurumi-ku, Yokohama, Kanagawa 230-0045, Japan; 5 Tokushima Breast Care Clinic, 4-7-7, Nakashimada-cho, Tokushima 770-0052, Japan; 6 Laboratory of Molecular Medicine, Human Genome Center, Institute of Medical Science, The University of Tokyo, 4-6-1 Shirokanedai, Minato-ku, Tokyo 108-8639, Japan

## Abstract

**Introduction:**

Chemotherapy-induced alopecia is one of the most common adverse events caused by conventional cytotoxic chemotherapy, yet there has been very little progress in the prevention or treatment of this side effect. Although this is not a life-threatening event, alopecia is very psychologically difficult for many women to manage. In order to improve the quality of life for these women, it is important to elucidate the molecular mechanisms of chemotherapy-induced alopecia and develop ways to effectively prevent and/or treat it. To identify the genetic risk factors associated with chemotherapy-induced alopecia, we conducted a genome-wide association study (GWAS) using DNA samples from breast cancer patients who were treated with chemotherapy.

**Methods:**

We performed a case-control association study of 303 individuals who developed grade 2 alopecia, and compared them with 880 breast cancer patients who did not show hair loss after being treated with conventional chemotherapy. In addition, we separately analyzed a subset of patients who received specific combination therapies by GWASs and applied the weighted genetic risk scoring (wGRS) system to investigate the cumulative effects of the associated SNPs.

**Results:**

We identified an SNP significantly associated with drug-induced grade 2 alopecia (rs3820706 in *CACNB4* (calcium channel voltage-dependent subunit beta 4) on 2q23, *P =* 8.13 × 10^-9^, OR = 3.71) and detected several SNPs that showed some suggestive associations by subgroup analyses. We also classified patients into four groups on the basis of wGRS analysis and found that patients who classified in the highest risk group showed 443 times higher risk of antimicrotubule agents-induced alopecia than the lowest risk group.

**Conclusions:**

Our study suggests several associated genes and should shed some light on the molecular mechanism of alopecia in chemotherapy-treated breast cancer patients and hopefully will contribute to development of interventions that will improve the quality of life (QOL) of cancer patients.

## Introduction

Breast cancer is the most common malignancy among women worldwide [1]. Although treatment of breast cancer has been significantly improved by the development of molecular-targeted drugs in the past few decades, a subset of patients do not receive benefit from these modalities [2,3]. Such patients and the majority of relapsed patients are treated with conventional cytotoxic chemotherapy that can often cause various adverse events including hair loss.

Hair loss (alopecia) is one of the most common side effects caused by chemotherapy in cancer patients, particularly in women with breast cancer. Although molecular-targeted drugs such as trastuzumab do not cause alopecia, these drugs are given together with other chemotherapeutic agents. Most of the cytotoxic agents cause alopecia, but the severity in individual patients and the incidence by the types of drugs are significantly different: more than 80% of patients treated with antimicrotubule agents, more than 60% of those with alkylating agents, 60 to 100% of those with topoisomerase inhibitors, and 10 to 50% of those with antimetabolite-based drugs experience severe alopecia [4]. It is also well known that the incidence and the severity are increased when patients are treated with a combination of multiple drugs rather than a single agent [4,5]. Usually, hair loss begins one to two weeks after the start of chemotherapy and a patient’s hair can be completely lost in a one- to two-month period. Hair starts to regrow after chemotherapy is completed or discontinued [6,7]. This drug-induced hair loss is not a life-threatening side effect, however, it can strongly influence cosmetic appearance and psychological stresses, and often affects the quality of life (QOL) of the patients [7]. Several studies have demonstrated that the majority of women patients are distressed due to treatment-related alopecia and that 8% of the women avoid chemotherapy because they are unwilling to deal with hair loss [7-10]. Moreover, one study reported that the hair loss was harder to manage than the loss of a breast in some patients [11].

It is known that there are three cycles during hair growth: anagen is the growth phase; catagen is the involuting or regressing phase; and telogen is the resting or quiescent phase [12,13]. It is thought that chemotherapeutic agents target highly proliferative hair matrix cells in the anagen phase, called the anagen effluvium [4,14], but the molecular mechanism is still largely unknown. Scalp cooling with cold air or liquid is the most widely used method since the 1970s to prevent or minimize drug-induced alopecia. However, it is not always effective and it is not easy to standardize the system of scalp cooling [4,15]. Since medications such as minoxidil or AS101, which are widely used for aging-related hair loss, failed to show any protective effect in the case of chemotherapy-induced alopecia [16-19], there is currently no good option to prevent or treat drug-induced alopecia.

In this study, we conducted a genome-wide association study (GWAS) using mono- or combination-chemotherapy-treated breast cancer cases to identify common genetic factors that are associated with drug-induced alopecia. We have identified some loci that are likely to be associated with increased risk of chemotherapy-induced alopecia. These results can provide new insight into the molecular mechanisms of hair loss induced by anticancer drugs and may contribute to development of drugs that can prevent or treat this emotionally devastating side effect.

## Methods

### Participants

All samples used in this study were obtained from the BioBank Japan located at the Institute of Medical Science at the University of Tokyo. The BioBank Japan project [20], which began in 2003, is a collaborative network of 66 hospitals in Japan [21]. The project achieved a collection of genomic DNA, serum, and clinical information from a total of 330,000 cases (200,000 patients) that had at least 1 of 47 defined diseases. Adverse drug reaction (ADR) information was collected from the patients’ medical records by medical coordinators. From the BioBank Japan, we selected 1,367 individuals who had been diagnosed with breast cancer and had received conventional chemotherapy. Of them, 303 patients had experienced grade 2 alopecia (ADR), 184 revealed grade 1 alopecia, and the remaining 880 patients were reported to have had no alopecia (non-ADR). Grade 2 alopecia is defined as complete hair loss, which is the most severe grade in this adverse reaction (National Cancer Institute Common Toxicity Criteria (NCI-CTC) version 3.0). In addition, samples from 23 breast cancer patients with grade 2 alopecia were collected at the Tokushima Breast Care Clinic to further verify the findings of the initial GWAS study; all of the 23 patients were treated with a combination therapy of docetaxel and cyclophosphamide. The detailed clinical information is summarized in Additional file 1. All participants provided written informed consent. This project was approved by the Institutional Review Board of the Institute of Medical Science, the University of Tokyo, and RIKEN Center for Genomic Medicine.

### Genotyping and quality control

For GWAS, all DNA samples were genotyped using Illumina Human OmniExpress BeadChip kits (Illumina, San Diego, CA, USA). Sample quality control was performed by identity-by-state clustering across all samples to evaluate cryptic relatedness for each sample and by use of principal component analysis to exclude genetically heterogeneous samples from further analysis. We applied SNP quality control by excluding SNPs with a call rate of <0.99, a *P* value of the Hardy-Weinberg equilibrium test of ≤1.0 × 10^-6^, and non-polymorphic SNPs in the dataset. Quantile-quantile (Q-Q) plots and lambda values, which were used for further evaluation of population substructure, were calculated between observed *P* value from Fisher’s exact test allelic model against expected *P* value. For genotyping of additional samples, we used the multiplex PCR-based Invader assay (Third Wave Technologies, Madison, WI, USA) as described previously [22].

### Statistical analysis

In the GWAS, Fisher’s exact test was applied to three genetic models: an allele frequency model, a dominant inheritance model, and a recessive inheritance model. SNPs were rank-ordered according to the lowest *P* value among the three models. Odds ratio (OR) and confidence intervals (CIs) were calculated for the allelic model using a non-risk allele or a non-risk genotype as a reference. A Manhattan plot was generated by using the minimum *P* value among three genetic models. For the combined analysis, the genotype count of the additional samples was added to that of the GWAS. All statistical analyses and plots were carried out using R statistical environment version 2.13.2 [23], and PLINK version 1.07 [24,25]. Haploview software was used for haplotype analysis, to draw the Manhattan plot and linkage disequilibrium (LD) map.

### Scoring system using weighted genetic risk score (wGRS)

The scoring analysis was performed by utilizing SNPs with *P* min of <1.0 × 10^-5^ after exclusion of SNPs that show strong LD (r^2^ >0.8) of each GWAS. wGRSs were calculated according to a method reported by De Jager *et al.*[26]. Briefly, we first determined the effect size of each SNP, calculated the cumulative genetic risk scores by multiplying the number of risk alleles for each SNP by its corresponding weight, and subsequently took the sum across the total number of SNPs that were taken into consideration of each GWAS set. We classified the genetic risk score into four different groups, which were created from the mean and standard deviation (SD) as follows: <mean −1 SD for group 1; mean −1 SD to average for group 2; average to mean +1 SD for group 3; >mean +1 SD for group 4. Odds ratio (OR), 95% confidence interval (CI), *P* value, sensitivity, and specificity were calculated using group 1 as reference.

## Results

### Genome-wide association for chemotherapy-induced alopecia in breast cancer

We performed a GWAS of 303 individuals who developed grade 2 alopecia, and compared them with 880 breast cancer patients who did not show any hair loss after being treated with conventional chemotherapy. The Q-Q plot and lambda (λ) value (λ <1.000) indicated no evidence of population stratification between the cases and controls we analyzed (Additional file 2). After the data was quality controlled, association analysis was carried out for 555,600 autosomal SNPs by Fisher’s exact test on the basis of three genetic models: allelic-effect, dominant-inheritance, and recessive-inheritance models. Among the SNPs analyzed in the GWAS, we identified a locus that reached genome-wide significance (rs3820706 near *CACNB4*, minimum *P =* 8.13 × 10^-9^, ORrec = 3.71, 95% CI: 2.24 to 6.15) and five additional loci that revealed suggestive association with chemotherapy-induced alopecia with a *P* value of <10^-6^ (Additional file 3 and Table 1). We further validated the top nine SNPs that revealed the smallest *P* value on the three loci in the GWAS result, using 23 additionally obtained alopecia cases. The combined analysis slightly improved the association with the rs3820706 locus (combined minimum *P =* 1.85 × 10^-9^, ORrec = 2.38, 95% CI: 1.44 to 3.93) and a nearby SNP rs16830728 (combined minimum *P =* 2.60 × 10^-8^, ORrec = 3.61, 95% CI: 2.17 to 5.98; Table 2). As these two SNPs are in strong LD with r^2^ of >0.8, we performed haplotype analysis, but the association was not as strong as those of single SNPs (Additional file 4 and Additional file 5).

**Table 1:** Summary of association results of the genome-wide association study

				** ADR**^**b**^			** Non-ADR**^**c**^			**RAF**		***P *** **value**				
**CHR**	**SNP**	**Gene**	**Allele 1/2 (risk)**	**11**	**12**	**22**	**11**	**12**	**22**	**ADR**	**Non-ADR**	**Allelic**	**Dominant**	**Recessive**	**OR**^**a**^	**95% CI**
2	rs3820706	*CACNB4*	A/G (G)	18	169	116	167	421	291	0.66	0.57	8.26E-05	1.07E-01	8.13E-09	3.71	(2.24-6.15)
2	rs6725180	*CACNB4*	A/C (C)	17	152	134	135	429	316	0.69	0.60	7.90E-05	1.11E-02	3.84E-06	3.05	(1.81-5.14)
8	rs16908658	*FAM135B*	G/A (G)	30	93	180	23	286	571	0.25	0.19	1.07E-03	9.68E-02	9.93E-07	4.09	(2.34-7.17)
10	rs7476422	*PCDH15*	T/G (G)	4	47	252	34	245	601	0.91	0.82	1.20E-07	3.77E-07	3.58E-02	2.17	(1.60-2.93)
10	rs857373	*PCDH15*	G/A (A)	5	55	243	43	255	581	0.89	0.81	5.16E-07	3.15E-06	1.11E-02	2.00	(1.51-2.66)
10	rs857392	*PCDH15*	G/A (A)	5	55	243	42	252	584	0.89	0.81	9.08E-07	5.95E-06	1.60E-02	1.97	(1.48-2.62)
10	rs1319836	*PCDH15*	C/T (T)	5	55	243	42	254	583	0.89	0.81	9.10E-07	4.34E-06	1.60E-02	1.98	(1.49-2.63)
10	rs7919725	*PCDH15*	A/G (G)	5	56	242	42	256	580	0.89	0.81	9.94E-07	4.68E-06	1.60E-02	1.97	(1.48-2.60)
10	rs857369	*PCDH15*	T/C (C)	1	32	270	18	178	684	0.94	0.88	2.29E-06	7.25E-06	5.87E-02	2.33	(1.60-3.39)
10	rs9416306	*PCDH15*	G/T (T)	1	32	270	18	178	682	0.94	0.88	2.29E-06	7.13E-06	5.88E-02	2.34	(1.61-3.39)
10	rs1219862	*PCDH15*	C/T (T)	2	31	270	17	182	681	0.94	0.88	2.73E-06	5.08E-06	1.85E-01	2.28	(1.58-3.30)
13	rs7318267	*FARP1*	C/T (T)	11	149	143	108	387	385	0.72	0.66	6.69E-03	3.15E-01	4.09E-06	3.71	(1.97-7.01)
13	rs2282048	*FARP1*	T/C (C)	11	148	144	107	387	386	0.72	0.66	5.72E-03	2.84E-01	6.24E-06	3.68	(1.95-6.93)
17	rs1530357	*LOC100506974*	A/G (A)	57	170	76	114	417	349	0.47	0.37	1.11E-05	4.29E-06	1.39E-02	1.96	(1.45-2.63)
17	rs1530361	*LOC100506974*	A/G (A)	53	165	85	99	408	372	0.45	0.35	8.83E-06	1.12E-05	7.04E-03	1.54	(1.27-1.86)
19	rs11666971	*LASS4*	G/A (G)	46	119	138	56	379	445	0.35	0.28	1.64E-03	1.43E-01	8.13E-06	2.63	(1.74-3.96)

^a^ORs and CIs are calculated according to the associated genetic model; ^b^individuals who developed grade 2 alopecia; ^c^individuals who did not developed any ADRs after chemotherapy. CHR, chromosome; SNP, single nucleotide polymorphism; ADR, adverse drug reaction; RAF, risk allele frequency; OR, odds ratio; CI, confidence interval.

**Table 2:** Summary of combined results of the genome-wide association study and additional genotyped data

						** ADR**^**c**^				** Non-ADR**^**d**^				***P *** **value**				
**SNP**	**CHR**	**Chromosome position**^**a**^	**Gene**	**Allele 1/2 (risk)**		**11**	**12**	**22**	**RAF**	**11**	**12**	**22**	**RAF**	**Allelic**	**Dominant**	**Recessive**	***P *** **min**	**OR**^**b **^**(95% CI)**
rs3820706	2	152957411	*CACNB4*	A/G	GWAS	18	169	116	0.66	167	421	291	0.57	8.26E-05	1.07E-01	8.13E-09	8.13E-09	3.71
																		(2.24-6.15)
				(G)	2nd	1	12	10	0.70	167	421	291	0.57	9.80E-02	3.70E-01	1.00E-01	9.80E-02	1.72
																		(0.91-3.25)
					Combine	19	181	126	0.66	167	421	291	0.57	3.16E-05	7.65E-02	1.85E-09	1.85E-09	2.38
																		(1.44-3.93)
rs16830728	2	152981335	*STAM2*	G/T	GWAS	17	163	123	0.68	153	422	304	0.59	1.11E-04	6.16E-02	7.24E-08	7.24E-08	3.54
																		(2.11-5.96)
				(T)	2nd	1	11	11	0.72	153	422	304	0.59	9.40E-02	1.91E-01	1.55E-01	9.40E-02	1.79
																		(0.94-3.43)
					Combine	18	174	134	0.68	153	422	304	0.59	3.49E-05	4.30E-02	2.60E-08	2.60E-08	3.61
																		(2.17-5.98)
rs7476422	10	56204291	*PCDH15*	T/G	GWAS	4	47	252	0.91	34	245	601	0.82	1.20E-07	3.77E-07	3.58E-02	1.20E-07	2.17
																		(1.60-2.93)
				(G)	2nd	0	7	16	0.85	34	245	601	0.82	8.45E-01	1.00E+00	1.00E+00	8.45E-01	1.21
																		(0.53-2.72)
					Combine	4	54	268	0.91	34	245	601	0.82	2.63E-07	1.15E-06	2.41E-02	2.63E-07	2.06
																		(1.54-2.75)

^a^On the basis of NCBI 36 genome assembly; ^b^ORs and CIs are calculated according to the associated genetic model; ^c^individuals who developed grade 2 alopecia; ^d^individuals who did not developed any ADRs after chemotherapy. The same controls were used in the GWAS and second stages analysis. SNP, single nucleotide polymorphism; CHR, chromosome; ADR, adverse drug reaction; RAF, risk allele frequency; *P* min, minimum *P* value; OR, odds ratio; CI, confidence interval.

### Association studies for drug subgroups and specific drugs

We also performed subgroup analyses for different types of chemotherapy, namely the CEF (cyclophosphamide + epirubicin +/− 5-FU)-treated and CAF (cyclophosphamide + doxorubicin +/− 5-FU)-treated groups. Detailed sample demographics are described in Additional file 1. In the GWAS of the CEF-treated group, genetic variants in the *ALOX5AP* gene on chromosome 13 were most significantly associated with chemotherapy-induced alopecia (rs3885907, minimum *P =* 1.38 × 10^-6^, OR = 2.66, 95% CI: 1.71 to 4.13). The GWAS analysis for the CAF-treated group identified SNP rs594206 located in an intronic region of *BCL9* on chromosome 1 to be most strongly associated (minimum *P =* 5.91 × 10^-7^, OR = 36.3, 95% CI: 4.58 to 287; Additional file 3 and Additional file 6). Although the *P* values for these variants did not exceed the genome-wide significance, it is notable that OR for the identified SNP for the CAF analysis is very large. In addition, we analyzed the association with antimicrotubule agents, paclitaxel monotherapy and docetaxel monotherapy because of their high incidence of alopecia, and found that rs1858231 (minimum *P =* 1.95 × 10^-6^, OR = 2.71, 95% CI: 1.79 to 4.12), rs11059635 (minimum *P =* 2.05 × 10^-7^, OR = 6.63, 95% CI: 2.95 to 14.9) and rs4262906 (minimum *P =* 6.62 × 10^-7^, OR = 4.36, 95% CI: 2.41 to 7.89) were most significantly associated, respectively (Additional file 6).

SNP rs3820706 on *CACNB4*, which showed the strongest association with chemotherapy-induced alopecia with the genome-wide significance in the analysis of all-combined samples, showed modest associations in all of the subgroup analyses (Additional file 7). Although the numbers of samples in these subgroup analyses were relatively limited, these data may provide fundamental information that will contribute to a better understanding of chemotherapy-induced alopecia.

### Scoring system for prediction of chemotherapy-induced alopecia

We then evaluated the cumulative effects of the candidate loci (SNPs showing *P* <10^-5^ in Table 1 and Additional file 6) using a weighted genetic risk scoring (wGRS) method [26]. We first selected eight SNPs from the GWAS of the combination of all samples and calculated wGRS. As shown in Additional file 8, only 17 of 190 patients belonging to group 1 showed severe hair loss (grade 2) while 54 of 82 patients in group 4 revealed it. Cumulative risk scores for the risk of drug-induced alopecia were calculated to be 4.44 in group 3 and 19.6 in group 4 (*P =* 3.44 × 10^-9^, 95% CI: 2.62 to 7.53; *P =* 1.44 × 10^-21^, 95% CI: 9.99 to 38.6, respectively), compared with patients in group 1.

**Figure 1:**
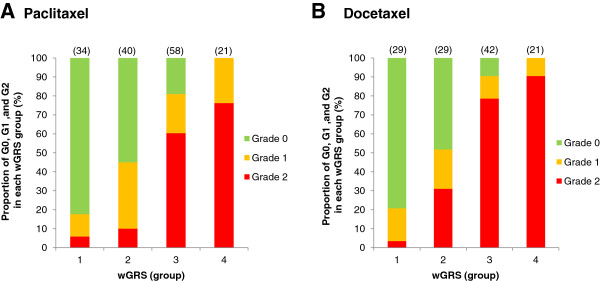
**The proportions of patients by alopecia grade in each weighted genomic risk score.** The proportions of patients who developed no adverse reaction (G0), grade 1 alopecia, or grade 2 alopecia in each of the weighted genomic risk score (wGRS) groups. The number in parentheses indicates the number of samples in each group. **(A)** paclitaxel monotherapy, **(B)** docetaxel monotherapy.

Similarly, in the subgroup analysis, an individual belonging to group 4 with the highest risk score in each of the CEF, CAF, antimicrotubules, paclitaxel, and docetaxel analyses was estimated to have 86.2 times, 891 times, 858 times, 1,680 times, and 441 times higher risk for the drug-related alopecia than those in group 1, respectively (Additional file 8). Due to the clinical importance of antimicrotubule agents (paclitaxel and docetaxel), which cause chemotherapy-induced alopecia at nearly 80% frequency, we further investigated the wGRS scoring method using cases with grade 1 alopecia. Interestingly, the association levels and odds ratios of patients with grade 1 alopecia induced by the antimicrotubule agents were intermediate, compared with those of grade 2 alopecia (Table 3). Not only antimicrotubule agents, but other subgroups (all, CEA or CEF) also showed similar results, and the association level of grade 1 was intermediate compared with grade 2. These results further support a possible association of these variants in alopecia development (Additional file 9). As shown in Figure 1, the proportion of grade 2 alopecia increased according to the increase of the wGRS score; for example, in the case of docetaxel, only one (3.4%) of the 29 patients in group 1 revealed grade 2 alopecia, while 52 (83%) of 63 patients belonging to groups 3 and 4 developed grade 2 alopecia. These results indicate that our scoring system may be applied to predict severe chemotherapy-induced alopecia and might provide useful information for better understanding of the hair-loss mechanism, even though further verification using an additional independent set(s) of samples is warranted.

**Table 3:** wGRS results of antimicrotubule agents, docetaxel, and paclitaxel-induced alopecia

**Cat**	**Score**	**G2**^**a**^	**G1**^**b**^	**G0**^**c**^	**%-G2**	**%-G1**	**%-G0**	** G2 vs. G0**			** G1 vs. G0**		
								**OR**^d*^	**95%CI**	***P *** **value**	**OR**^d*^	**95%CI**	***P *** **value**
**Antimicrotubule (6 SNPs)**													
1	<5.56	2	7	34	0.05	0.16	0.79		Ref			Ref	
2	5.56-7.60	25	20	50	0.26	0.21	0.53	8.50	1.89-38.3	1.66E-03	1.94	0.74-1.42	2.52E-01
3	7.60-9.63	65	17	19	0.64	0.17	0.19	58.2	12.8-265	4.93E-14	4.35	1.53-12.4	6.42E-03
4	>9.63	26	6	1	0.79	0.18	0.03	442	38.0-5140	2.71E-14	29.1	3.02-282	8.39E-04
	**Total**	**118**	**50**	**104**									
**Docetaxel (4 SNPs)**													
1	<2.26	1	5	23	0.03	0.17	0.79		Ref			Ref	
2	2.26-4.70	9	6	14	0.31	0.21	0.48	14.8	1.69-130	4.39E-03	1.97	0.51-7.68	4.88E-01
3	4.70-7.15	33	5	4	0.79	0.12	0.10	190	19.9-1810	1.01E-11	5.75	1.12-29.4	4.08E-02
4	>7.15	19	2	0	0.90	0.10	0.00	611	23.5-15900	2.50E-11	21.4	0.89-511	4.83E-02
	**Total**	**62**	**18**	**41**									
**Paclitaxel (7 SNPs)**													
1	<3.24	2	4	28	0.06	0.12	0.82		Ref			Ref	
2	3.24-7.48	4	14	22	0.10	0.35	0.55	2.55	0.43-15.2	4.01E-01	4.46	1.28-1.92	2.60E-02
3	7.48-11.7	35	12	11	0.60	0.21	0.19	44.6	9.12-218	9.55E-10	7.64	2.02-28.9	2.30E-03
4	>11.7	16	5	0	0.76	0.24	0.00	376	17.0-8320	1.54E-10	69.7	3.26-1490	2.89E-04
	**Total**	**57**	**35**	**61**									

^a^Individuals who developed grade 2 alopecia; ^b^individuals who developed grade 1 alopecia; ^c^individuals who did not developed any ADRs after chemotherapy; ^d^ORs and CIs are calculated using category (group) 1 as reference. *OR calculated after Haldane’s correction: adding 0.5 to all the cells of a contingency table if any of the cell expectations would cause a division by zero error. Cat, category; OR, odds ratio; CI, confidence interval; Ref, reference.

Finally, we simulated the sample number that is required to verify our scoring system. In BioBank Japan, a total of 279 patients received antimicrotubule agents (paclitaxel and/or docetaxel). Among them, 119 (43%) patients developed grade 2 alopecia, 55 (20%) developed grade 1 alopecia and 105 (37%) did not show any adverse events. Among 156 patients who received paclitaxel monotherapy, 57 (37%) developed grade 2 alopecia, 36 (23%) developed grade 1 alopecia and 63 (40%) did not develop any adverse reactions. When we assume that 100 patients who receive antimicrotubule agents (or paclitaxel monotherapy) are registered, the incidences of alopecia are estimated as shown in Table 4. If we categorize the patients by wGRS according to the data in Table 3, 100 additional patients should provide the sufficient statistical power to verify our results with *P* value of <0.01. Even if two individuals in each of groups 1 and 4 are not correctly predicted, the calculated *P* value is still 0.001 by Fisher’s exact test.

**Table 4:** Estimation of required sample number for verification

	**Cat**	**G2**	**G0**	**OR**	**95% CI**	***P *** value*
**Antimicrotubule (paclitaxel and docetaxel) (N = 100)**						
	1	1	12		Ref	
	2	9	18	6.00	0.67-53.7	1.24E-01
	3	24	7	41.1	4.53-374	2.48E-05
	4	9	1	108	5.92-1970	1.15E-04
	**Total**	43	38			
**Paclitaxel (N = 100)**						
	1	1	18		Ref	
	2	3	15	3.60	0.34-38.3	3.40E-01
	3	23	7	59.1	6.66-525	8.02E-07
	4	10	0	259	9.66-6950	5.49E-07
	**Total**	37	40			

*P values are calculated by Fisher’s exact test. Cat, category; OR, odds ratio; CI, confidence interval; Ref, reference.

## Discussion

Recent pharmacogenomics studies focus on prediction of drug response as well as the risk assessment of toxic events due to administration of drugs. Whole-genome association studies have been proven to be a powerful strategy to identify genetic factor(s) associated with various adverse reactions caused by certain drugs. In this study, we conducted the first GWAS for chemotherapy-induced alopecia in Japanese breast cancer patients, and identified one locus including two SNPs, rs3820706 on chromosome 2q23 and its nearby SNP rs16830728, which showed a strong association with genome-wide significance, and found several SNPs showing suggestive associations.

SNP rs3820706 is located near a gene encoding calcium channel voltage-dependent subunit beta 4 (*CACNB4*), a member of a beta subunit family of the voltage-dependent calcium channel (VDCC) complex. Calcium (Ca^2+^) functions as a second messenger in many cellular signal transduction pathways such as cell proliferation and apoptosis. When VDCC is activated it depolarizes membrane potentials, it allows Ca^2+^ to enter into cells [27]. We are not aware of any previous reports indicating that there is a relationship between the Ca^2+^ channel and alopecia. However, a potassium channel opener, minoxidil, was approved for the treatment of alopecia by the US FDA in 1988 [28] and has proven to be effective in a subset of alopecia patients. Although the mode of action of minoxidil is still not well known, the clinical outcome implies the involvement of ion channels for K^+^ and probably Ca^2+^ in the pathogenesis of alopecia. Intriguingly, the second most significantly associated locus that we found in our study is a region containing the *PCDH15* gene on chromosome 10. *PCDH15* encodes a protocadherin-related protein, which is involved in calcium-dependent cell-cell adhesion. Additionally, among the 70 loci in the top 100 SNPs found in our GWAS study, five loci are implicated to be ion channels or proteins related to ion channels (data not shown). Ion channels have shown to have important roles not only in cell maintenance but also in stem/progenitor cells [29]. Because cytotoxic agents damage the proliferating progenitor cells in the hair matrix [13], we suspect that several ion channels might be involved in chemotherapy-induced alopecia and be promising targets for development of novel treatments.

However, since rs3820706 is strongly linked to rs16830728, which is located within a gene encoding a signal transducing adaptor molecule 2 (*STAM2*), we cannot exclude the possibility that *STAM2* is a candidate gene for chemotherapy-induced alopecia. STAM2 is a member of the STAM family, which is an adaptor protein involved in the downstream signaling of cytokine receptors that contain an SH3 domain and the immunoreceptor tyrosine-based activation motif (ITAM). STAM2 is involved in the signaling through GM-SCF and IL-2 stimulation, and has a crucial role in T cell development [30,31]. As most studies of STAM2 focused on immune cells, its functions in other cell types like hair follicle cells are not fully understood.

In addition, we performed subgroup analyses in which we identified multiple loci that might be associated with drug-induced alopecia. rs3885907, which was most significantly associated in CEF-treated patients, was located in an intron of *ALOX5AP*. *ALOX5AP*, arachidonate 5-lipoxygenase-activating protein, is related to the inflammatory responses and possibly to vascular diseases [32,33]. Detailed biological mechanisms in hair growth cycle are not well characterized, but one paper reported involvement of the *ALOX5AP* upregulation in scarring alopecia [34]. According to GWAS, for alopecia areata [35] that identified genes related in both innate and adaptive immunity, inflammatory or immune responses seem to be important in alopecia development. The mechanisms of hair loss in alopecia areata and in drug-induced alopecia may not be same, but our result suggests a possible relationship of the immune response with chemotherapy-induced alopecia.

A SNP in the *BCL9* gene was most significantly associated with hair loss in the CAF-treated group with very high OR of 36.3. The *BCL9* gene encodes B-cell lymphoma 9 which was reported to interact with β-catenin. The β-catenin signaling pathway is involved in hair follicle morphogenesis during embryogenesis and, interestingly, hair is completely lost when β-catenin is depleted even after hair follicles have been formed [36,37]. Similarly, *CDH7*, one of the cadherin family members, showed an association with severe hair loss in the CAF-treated group with high OR of 32.5. This cadherin has been reported to be expressed in hair follicles and regulate hair growth [38,39]. These results, in combination with our GWAS results, imply possible roles for BCL9 and CDH7 in chemotherapy-induced alopecia. If so, these two molecules as well as CACNB4 and other ion channel proteins could be promising targets for the development of new treatments. However, further validation is still needed.

Our approach of using retrospective BioBank samples is not ideal for addressing this type of clinical problem and certainly a prospective analysis with well-defined clinical information would reduce the possibility of false-positive and false-negative results. However, considering the rapid progress of drug development or new combination therapies in recent years, it may not be wise to spend lots of effort, time and budget to do a prospective study, because the investigated regimen may not be used years later when the research results come out. One of the ways to effectively use the data and samples from the retrospective study is shown by the application of our wGRS system. The wGRS system indicated cumulative effect of multiple genetic variants for alopecia prediction. For example, the patients in group 4 who received paclitaxel showed 376 times increased risk of alopecia, compared with those belonging to group 1. Similarly, the patients in group 4 who received docetaxel showed 611 times higher risk of alopecia than those belonging to group 1. We understand the disadvantages and pitfalls of the retrospective design for the pharmacogenomics study such as the higher risk of false results. However, considering the very high OR obtained by the wGRS system, the advantage of this approach is that we are able to verify the results by using a relatively small number of additional prospective samples. We simulated the sample size needed to verify our results, as shown in Table 4, and suggest that the statistical power should be sufficient to validate with this small number of samples. We recognize that the clinical utility for this wGRS may not be as high as in other studies looking at life-threatening adverse events. However, identification of genetic factors associated with drug-induced hair loss should be the first step to understand the molecular mechanism and to contribute to the development of new drugs to prevent or treat alopecia.

For many years, breast cancer patients have had to accept the psychologically stressful side effect of alopecia caused by cytotoxic chemotherapies. It is known that a subset of patients will refuse to have chemotherapy because they do not want to lose their hair and therefore may lose the opportunity to receive the benefit of the chemotherapy and a chance to be cured of their disease. The QOL of these patients is extremely important and we believe it is urgent that we work to develop new treatment or prevention strategies to manage chemotherapy-induced alopecia. Although further validation of our findings is required, our study identified some significant molecular alterations in genes such as ion channel-related genes and genes in the β-catenin signaling pathway. We welcome other groups to examine and validate our results and hope these findings will contribute to the development of interventions that will improve the quality of life (QOL) of breast cancer patients.

## Conclusions

In summary, we identified strongly associated genetic variants near gene *CACNB4* and several suggestively associated SNPs with chemotherapy-induced alopecia in breast cancer patients. These results provide new information of the pathogenesis of chemotherapy-induced alopecia.

## Abbreviations

ADR: Adverse drug reaction; CAF: Cyclophosphamide + doxorubicin +/− 5-FU; CEF: Cyclophosphamide + epirubicin +/− 5-; CI: Confidence interval; GWAS: Genome-wide association study; LD: Linkage disequilibrium; OR: Odds ratio; QOL: Quality of life; QQ plot: Quantile-quantile plot; SD: Standard deviation; SNP: Single nucleotide polymorphism; wGRS: Weighted genomic risk score.

## Competing interests

The authors declare that they have no competing interests.

## Authors’ contributions

YN planned and supervised the study and obtained funding. SC and SKL designed the experiments and performed the GWAS and combined analysis. SKL performed the wGRS and statistical analysis. HZ and MS collected additional samples and medical information. MK genotyped all BioBank Japan samples. AT performed sample quality control. YN, SC and SKL wrote the manuscript. All authors revised and approved the manuscript for publication.

## Supplementary Material

Additional file 1: Table S1 Patients’ characteristics. Click here for file

Additional file 2 Quantile-quantile plot of the genome-wide association study. Click here for file

Additional file 3 Manhattan plot of the genome-wide association study for chemotherapy-induced alopecia in breast cancer. Click here for file

Additional file 4 Haplotype analysis. Click here for file

Additional file 5: Table S2 Haplotype analysis of two SNPs. Click here for file

Additional file 6: Table S3 Summary of genome-wide association study for chemotherapy-induced alopecia with each drug subgroup (*P* <10-6). Click here for file

Additional file 7: Table S4 Association of rs3820706 in subgroups. Click here for file

Additional file 8: Table S5 Weighted genomic risk score of each genome-wide association study for chemotherapy-induced alopecia. Click here for file

Additional file 9: Table S6 Weighted genomic risk score results of all, CAF- and CEF-induced alopecia. Click here for file
