# Supplementary material for: A genome-wide association study of chemotherapy-induced alopecia in breast cancer patients
Source: Breast Cancer Res. 2013 Sep 11;15(5):R81. doi: 10.1186/bcr3475 (PMC3978764; doi:10.1186/bcr3475)
Supplement: Additional file 1: Table S1 — Patients’ characteristics. [file bcr3475-S1.pdf]

Supplementary Table 1   Patients' characteristics

Stage	Platform	Sourse	Age (Mean)	No. of samples
GWAS_All[b]				
ADR[c]	Illimina Human OmniExpress	BioBank Japan	53.7	303
Non-ADR[d]	Illimina Human OmniExpress	BioBank Japan	58.0	880
Combined analysis[a]				
ADR	Invader assay	Tokushima Breast Care Clinic	51.4	23

[a]We collected case sample only. All patients received combination of docetaxel and cyclophosphamide

[b]Most of the patients received combination therapy

[c]Individuals who developed grade 2 alopecia

[d]Individuals who did not developed any ADRs after chemotherapy

Category	Grade2*	Grade1**	Grade0***
**All**	303	184	880
**Age (Mean)**	53.7	53.4	58.0
**Subgroup analysis**			
Cyclophosphamide + Epirubicin +/- 5FU (CEF)	116	44	108
Cyclophosphamide + Doxorubicin +/- 5FU (CAF)	64	33	27
Anti-microtubules treatment	119	55	105
Paclitaxel monotherapy	57	36	63
Doxorubicin monotherapy	62	19	42

* Individuals with grade 2 alopecia indicate complete hair loss after receiving chemotherapeutic agents

** Individuals with grade 1 alopecia indicate partial hair loss after receiving chemotherapeutic agents

** Individuals with grade 0 alopecia indicate no incidence of hair loss loss after receiving chemotherapeutic agents
