# Supplementary material for: A genome-wide association study of chemotherapy-induced alopecia in breast cancer patients
Source: Breast Cancer Res. 2013 Sep 11;15(5):R81. doi: 10.1186/bcr3475 (PMC3978764; doi:10.1186/bcr3475)

Additional file 2 Q-Q plot of GWAS

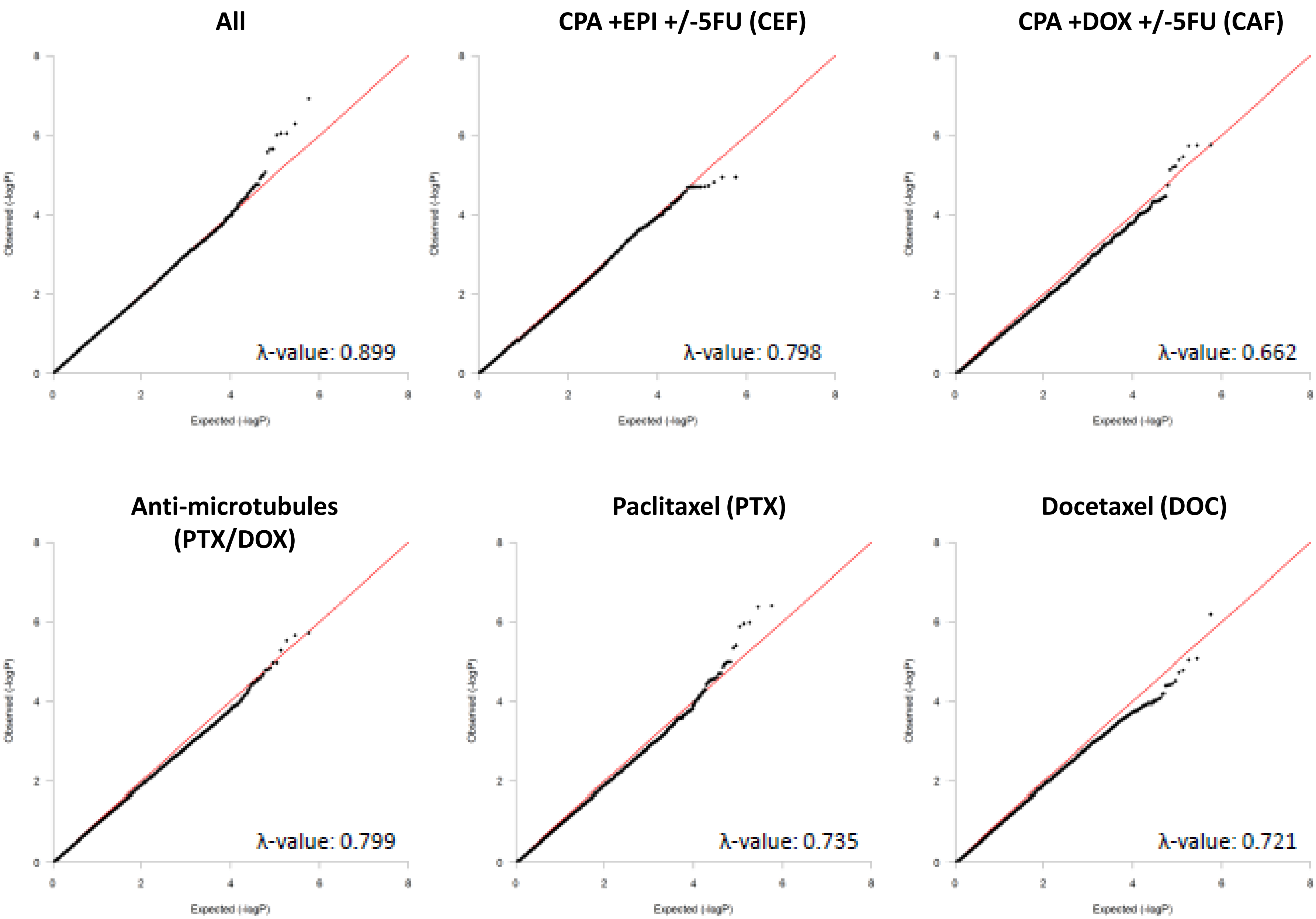

Supplement: Additional file 2 — Quantile-quantile plot of the genome-wide association study. [file bcr3475-S2.pdf]
