# Supplementary material for: A genome-wide association study of chemotherapy-induced alopecia in breast cancer patients
Source: Breast Cancer Res. 2013 Sep 11;15(5):R81. doi: 10.1186/bcr3475 (PMC3978764; doi:10.1186/bcr3475)

Additional file 3 Manhattan plot of GWAS for chemotherapy-induced alopecia in breast cancer

All

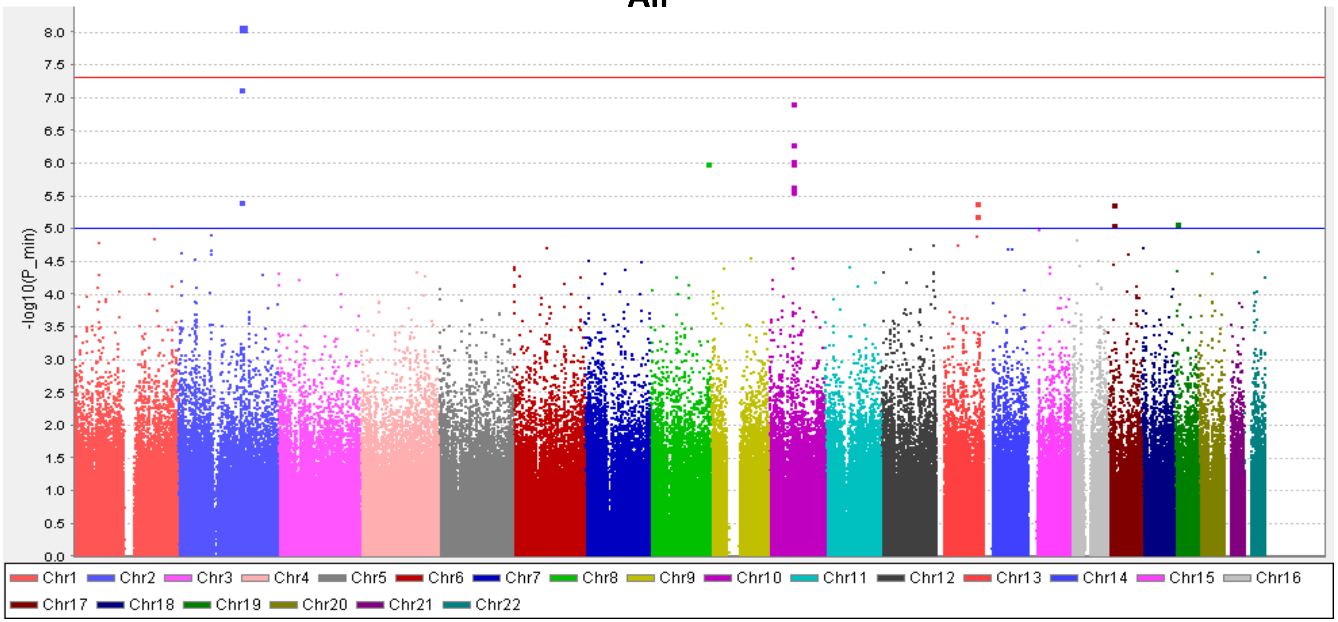

CPA +EPI +/-5FU (CEF)

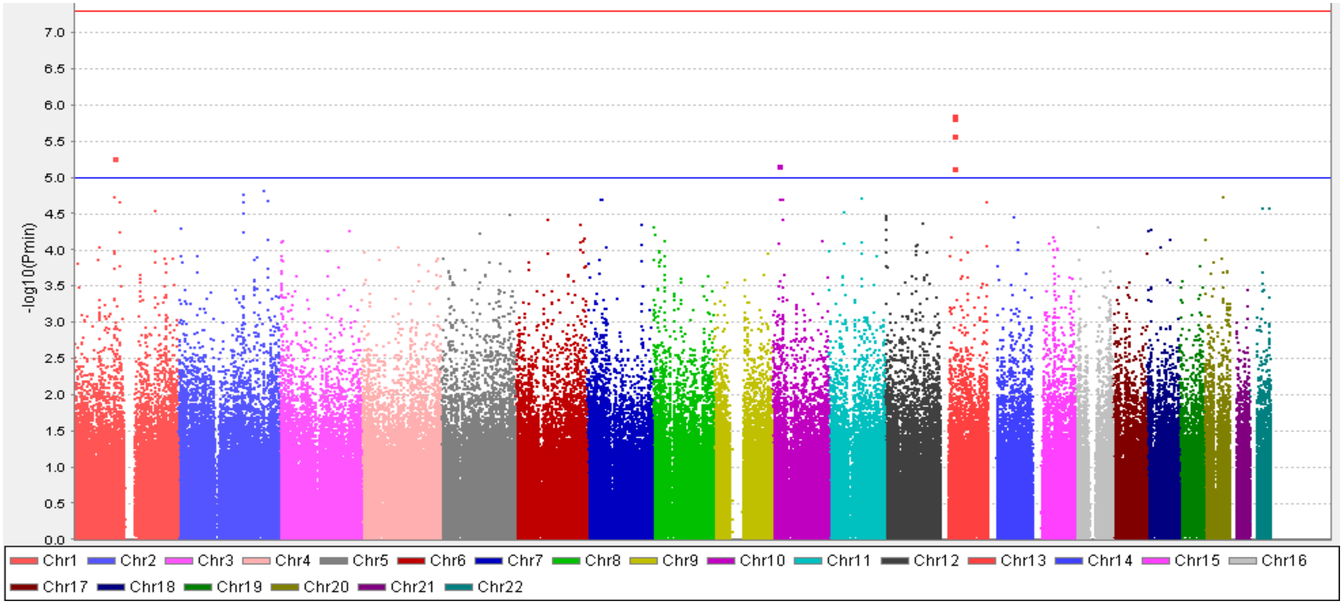

CPA +DOX +/-5FU (CAF)

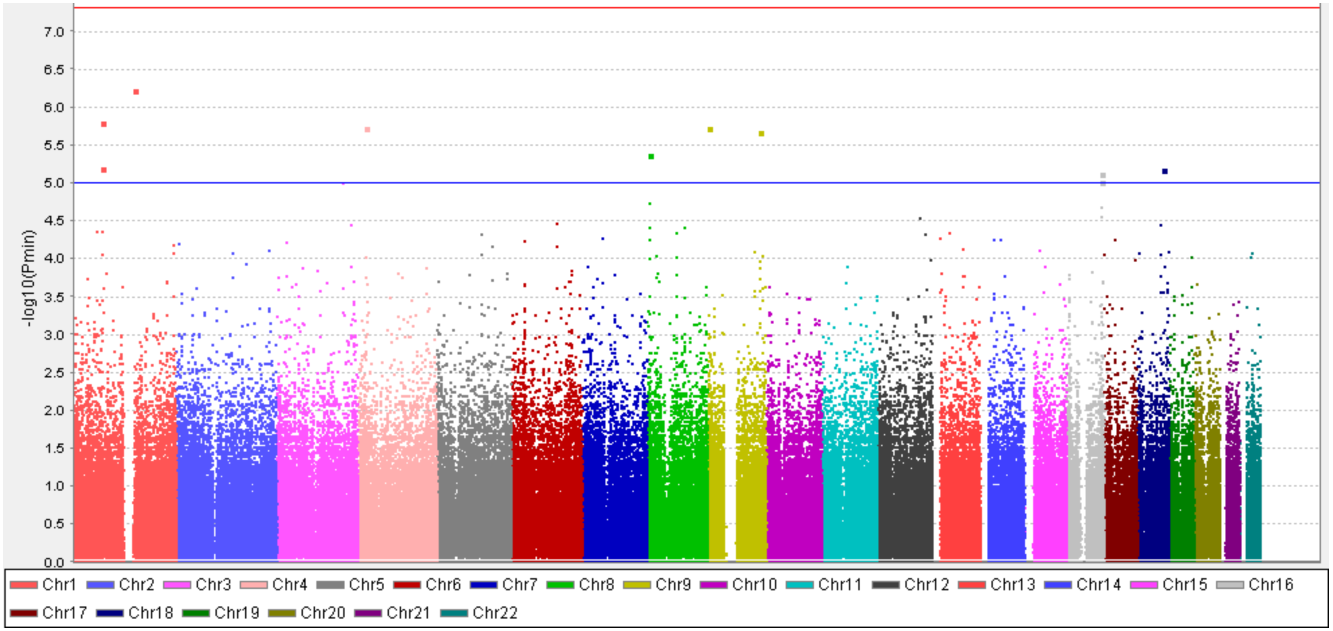

Anti-microtubules (PTX/DOC)

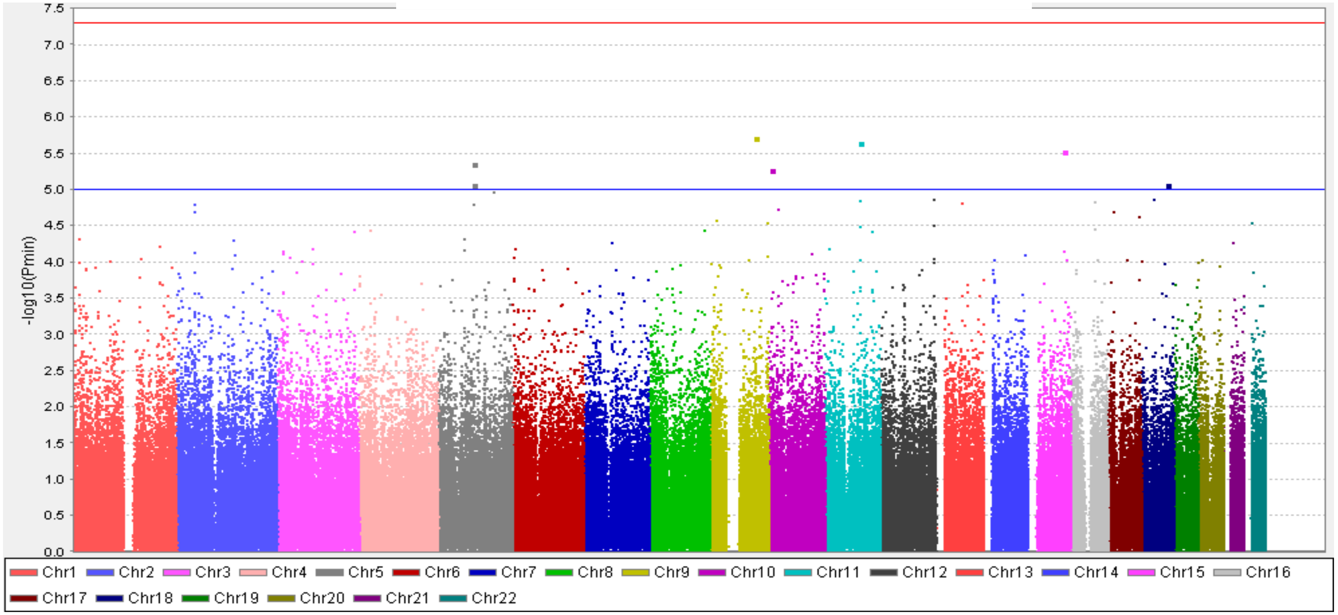

Paclitaxel (PTX)

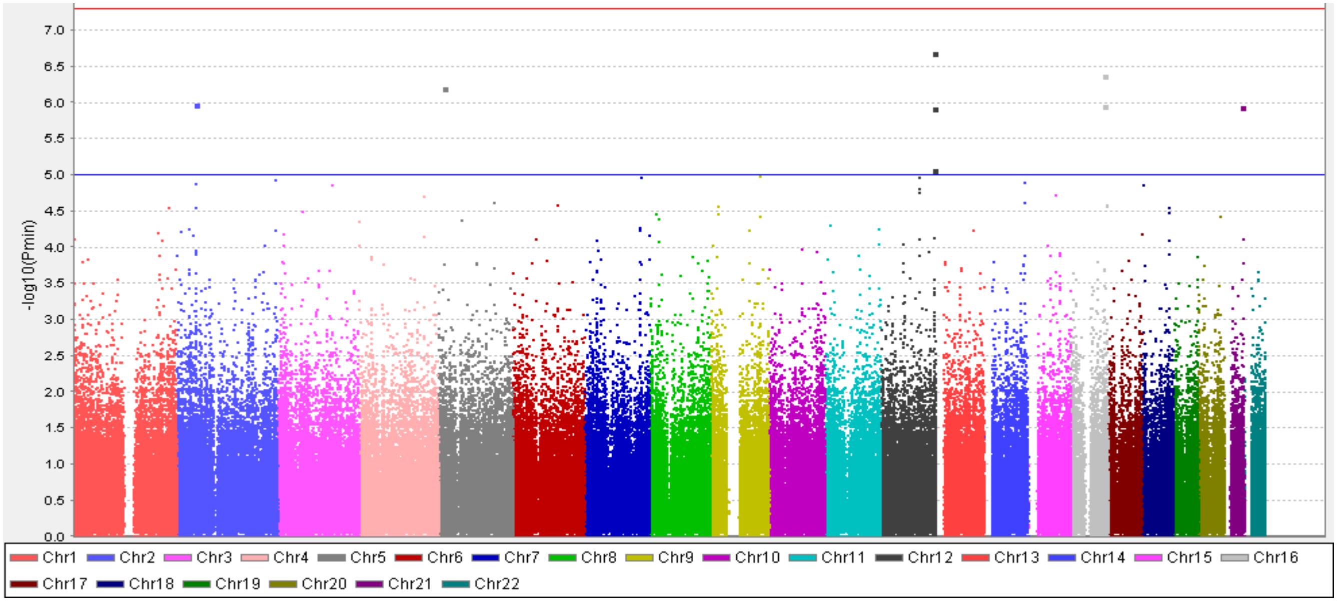

Docetaxel (DOC)

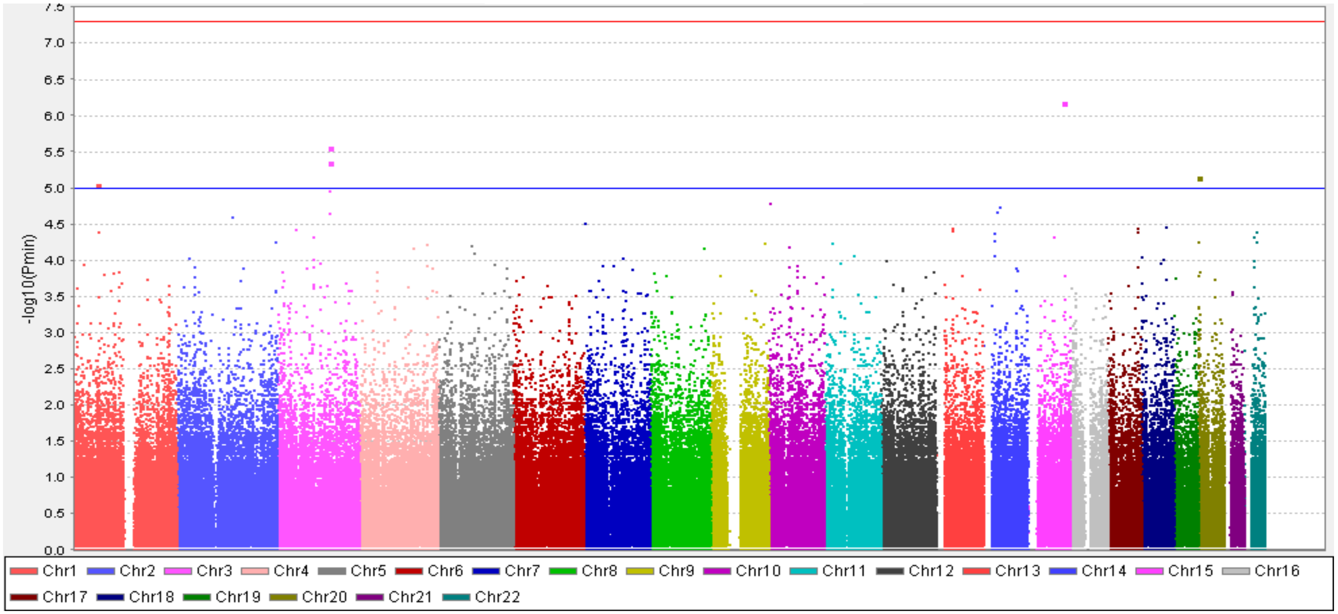

Supplement: Additional file 3 — Manhattan plot of the genome-wide association study for chemotherapy-induced alopecia in breast cancer. [file bcr3475-S3.pdf]
