# Supplementary material for: A genome-wide association study of chemotherapy-induced alopecia in breast cancer patients
Source: Breast Cancer Res. 2013 Sep 11;15(5):R81. doi: 10.1186/bcr3475 (PMC3978764; doi:10.1186/bcr3475)
Supplement: Additional file 5: Table S2 — Haplotype analysis of two SNPs. [file bcr3475-S5.pdf]

Supplementary Table 2 Haplotype analysis of 2 SNPs

CHR	SNP	BP	A1	A2	MAF		P-value				OR	95% CI	Gene	rel.loci
					ADR[a]	Non-ADR[b]	allelic	dominant	recessive	minimum				
2	rs3820706	152957411	A	G	0.34	0.43	8.26E-05	1.07E-01	8.13E-09	8.13E-09	1.47	(1.21-1.79)	CACNB4	-1818
2	rs16830728	152981335	G	T	0.33	0.41	1.11E-04	6.16E-02	7.24E-08	7.24E-08	1.47	(1.21-1.78)	STAM2	0

Haplotype/SNP allele(s)	Haplotype frequencies	ADR, Non-ADR frequencies	P value	OR*	Effect
GT	0.58	0.65, 0.37	5.91E-05	1.48	Risk
AG	0.38	0.32, 0.40	2.00E-04	0.69	Protective
AT	0.03	0.02, 0.03	0.571	0.84	Protective
GG	0.01	0.01, 0.01	0.6	0.77	Protective

SNP, single nucleotide polymorphism; ADR, adverse drug reaction; OR, odds ratio.

*ORs are calculated using the nonrisk haplotype as reference

[a]Individuals who developed grade 2 alopecia.

[b]Individuals who did not developed any ADRs after chemotherapy.
