# Supplementary material for: A genome-wide association study of chemotherapy-induced alopecia in breast cancer patients
Source: Breast Cancer Res. 2013 Sep 11;15(5):R81. doi: 10.1186/bcr3475 (PMC3978764; doi:10.1186/bcr3475)
Supplement: Additional file 6: Table S3 — Summary of genome-wide association study for chemotherapy-induced alopecia with each drug subgroup (P <10-6). [file bcr3475-S6.pdf]

Supplementary Table 3 Summary of GWAS for chemotherapy-induced alopecia with each drug subgroups ($P < 10^{-6}$)

Drug/drug subgroup	CHR	SNP	Gene	Allele 1/2 (risk)	RAF		P-value					
					ADR[b]	Non-ADR[c]	Allelic	Dominant	Recessive	Pmin	OR[a]	95% CI
CEF	13	rs3885907	*ALOX5AP*	G/T (G)	0.36	0.18	1.17E-05	1.38E-06	1.70E-01	1.38E-06	2.66	(1.71-4.13)
	13	rs9508835	*ALOX5AP*	T/G (T)	0.30	0.13	1.16E-05	1.52E-06	4.48E-01	1.52E-06	2.90	(1.78-4.72)
	13	rs10162089	*ALOX5AP*	T/C (T)	0.35	0.18	4.26E-05	2.66E-06	3.78E-01	2.66E-06	2.51	(1.62-3.91)
	1	rs4474258	*LOC100130235*	G/A (A)	0.58	0.43	1.21E-03	3.45E-01	5.29E-06	5.29E-06	1.89	(1.29-2.75)
	10	rs7089227	*PRPF18*	G/T (G)	0.58	0.41	3.34E-04	6.77E-06	2.80E-01	6.77E-06	1.99	(1.37-2.90)
	13	rs4075692	*ALOX5AP*	A/G (A)	0.31	0.15	5.08E-05	7.48E-06	4.48E-01	7.48E-06	2.59	(1.62-4.13)
	13	rs9671124	*ALOX5AP*	T/C (T)	0.31	0.15	5.08E-05	7.48E-06	4.48E-01	7.48E-06	2.59	(1.62-4.13)
CAF	1	rs594206	*BCL9*	G/A (A)	0.99	0.78	1.81E-06	5.91E-07	1.00E+00	5.91E-07	36.3	(4.58-287)
	1	rs672203	*CTH*	G/A (A)	0.85	0.52	7.68E-06	1.62E-06	2.41E-02	1.62E-06	5.33	(2.59-11.0)
	9	rs6475600	*SMARCA2*	T/C (C)	0.93	0.63	1.86E-06	2.53E-06	7.66E-02	1.86E-06	7.78	(3.25-18.6)
	4	rs6845621	*LCORL*	C/T (C)	0.59	0.20	1.91E-06	3.47E-06	1.58E-02	1.91E-06	5.53	(2.61-11.7)
	9	rs10818894	*DENND1A*	A/G (G)	0.96	0.70	3.59E-06	2.16E-06	2.97E-01	2.16E-06	10.4	(3.56-30.1)
	8	rs10112481	*LOC100129861*	A/C (A)	0.59	0.22	4.30E-06	8.72E-04	5.26E-05	4.30E-06	5.12	(2.46-10.6)
	1	rs8179319	*CTH*	A/G (G)	0.84	0.50	6.32E-06	6.92E-06	2.26E-02	6.32E-06	5.09	(2.51-10.4)
	18	rs637644	*CDH7*	A/G (G)	0.99	0.80	6.62E-06	4.80E-05	8.57E-02	6.62E-06	32.5	(4.08-259)
	16	rs11644424	*CDH13*	G/T (T)	0.73	0.44	3.82E-04	7.58E-06	3.30E-01	7.58E-06	3.32	(1.71-6.44)
	16	rs11860092	*CDH13*	G/A (A)	0.71	0.44	1.25E-03	9.66E-06	4.42E-01	9.66E-06	3.07	(1.59-5.94)
Anti-microtubule[d]	9	rs1858231	*ZNF462*	C/T (C)	0.43	0.22	1.95E-06	2.35E-05	6.22E-05	1.95E-06	2.71	(1.79-4.12)
	11	rs1870323	*ME3*	C/T (T)	0.84	0.64	2.23E-06	1.59E-05	8.06E-04	2.23E-06	2.92	(1.87-4.57)
	15	rs4262906	*KLHL25*	C/T (C)	0.57	0.35	3.04E-06	1.99E-04	4.41E-04	3.04E-06	2.50	(1.71-3.67)
	5	rs2471042	*RPL10AP9*	T/C (T)	0.40	0.21	1.60E-05	4.47E-06	9.07E-02	4.47E-06	2.51	(1.64-3.81)
	10	rs11255615	*LOC100507143*	G/A (G)	0.59	0.38	5.34E-06	9.77E-05	4.18E-04	5.34E-06	2.40	(1.65-3.53)
	5	rs1493354	*RPL10AP9*	T/C (T)	0.40	0.22	4.37E-05	8.53E-06	1.58E-01	8.53E-06	2.37	(1.56-3.60)
	5	rs13161175	*RPL10AP9*	A/C (A)	0.40	0.21	2.66E-05	8.53E-06	9.07E-02	8.53E-06	2.44	(1.60-3.71)
	18	rs2032224	*SERPINB5*	C/A (A)	0.78	0.62	2.71E-04	3.19E-02	8.75E-06	8.75E-06	2.16	(1.42-3.27)
Paclitaxel_mono	12	rs11059635	*TMEM132C*	A/G (G)	0.93	0.67	3.91E-07	2.05E-07	1.21E-01	2.05E-07	6.63	(2.95-14.9)
	16	rs3844412	*CDH13*	A/G (A)	0.40	0.11	4.20E-07	4.42E-07	9.94E-03	4.20E-07	5.22	(2.67-10.2)
	5	rs163818	*TRIO*	C/T (C)	0.22	0.02	1.35E-06	6.40E-07	4.75E-01	6.40E-07	11.5	(3.37-39.3)
	2	rs698813	*C2orf34*	T/C (C)	0.83	0.52	1.06E-06	1.08E-04	1.23E-04	1.06E-06	4.27	(2.35-7.76)
	16	rs4783238	*CDH13*	A/C (A)	0.40	0.12	1.12E-06	3.13E-06	4.44E-03	1.12E-06	4.83	(2.50-9.31)
	21	rs2838088	*NCRNA00111*	A/G (A)	0.38	0.12	4.04E-06	1.15E-06	1.01E-01	1.15E-06	4.48	(2.32-8.66)

	12	rs11059618	*TMEM132C*	G/A (A)	0.89	0.63	4.63E-06	1.18E-06	1.20E-01	1.18E-06	4.58	(2.32-9.07)
	12	rs10773519	*TMEM132C*	G/A (A)	0.83	0.58	1.98E-05	8.41E-06	6.39E-02	8.41E-06	3.63	(1.98-6.66)
Docetaxel_mono	15	rs4262906	*KLHL25*	C/T (C)	0.66	0.31	6.62E-07	1.72E-04	3.34E-04	6.62E-07	4.36	(2.41-7.89)
	3	rs4234284	*C3orf56*	T/C (T)	0.47	0.23	4.32E-04	2.78E-06	1.00E+00	2.78E-06	3.01	(1.62-5.60)
	3	rs13095826	*C3orf56*	G/T (G)	0.48	0.24	7.57E-04	4.49E-06	1.00E+00	4.49E-06	2.91	(1.57-5.36)
	20	rs6117615	*C20orf54*	C/T (C)	0.55	0.24	8.53E-06	7.14E-06	2.77E-02	7.14E-06	3.89	(2.10-7.18)
	1	rs10443215	*LOC100507652*	C/T (C)	0.26	0.04	9.21E-06	1.01E-05	2.71E-01	9.21E-06	9.39	(2.77-31.8)

CEF, cyclophosphamide+epirubicin+/-5FU; CAF, cyclophosphamide+doxorubicin+/-5FU; CHR, chromosome; SNP, single nucleotide polymorphism; ADR, adverse drug reaction; RAF, risk allele frequency; OR, odds ratio; CI, confidence interval.

[a]ORs and Cis are calculated using the nonrisk genotyope as reference.

[b]Individuals who developed grade 2 alopecia.

[c]Individuals who did not developed any ADRs after chemotherapy.

[d]Paclitaxel and docetaxel administrated samples.
