# Supplementary material for: A genome-wide association study of chemotherapy-induced alopecia in breast cancer patients
Source: Breast Cancer Res. 2013 Sep 11;15(5):R81. doi: 10.1186/bcr3475 (PMC3978764; doi:10.1186/bcr3475)
Supplement: Additional file 7: Table S4 — Association of rs3820706 in subgroups. [file bcr3475-S7.pdf]

Supplementary Table 4 Association of rs3820706 in subgroups

						RAF		P-value					
CHR	SNP	Gene	A1	A2	Subgroup	ADR[b]	Non-ADR[c]	allelic	dominant	recessive	minimum	OR[a]	95% CI
2	rs3820706	*CACNB4*	A	G	ALL	0.66	0.57	8.26E-05	1.07E-01	8.13E-09	8.13E-09	1.47	(1.21-1.79)
					CEF	0.64	0.51	5.45E-03	1.16E-01	1.10E-03	1.10E-03	1.73	(1.19-2.53)
					CAF	0.67	0.54	9.45E-02	6.39E-01	1.41E-02	1.41E-02	1.76	(0.92-3.38)
					Anti-microtubule[d]	0.63	0.56	1.02E-01	2.51E-01	9.93E-02	9.93E-02	1.38	(0.94-2.02)
					DOC mono	0.67	0.56	1.12E-01	1.49E-01	3.09E-01	1.12E-01	1.59	(0.90-2.82)
					PTX mono	0.60	0.56	6.01E-01	1.00E+00	3.07E-01	3.07E-01	1.18	(0.71-1.98)

CEF, cyclophosphamide+epirubicin+/-5FU; CAF, cyclophosphamide+doxorubicin+/-5FU; CHR, chromosome; SNP, single nucleotide polymorphism; ADR, adverse drug reaction; RAF, risk allele frequency; OR, odds ratio; CI, confidence interval.

[a]ORs and Cis are calculated using the nonrisk genotyope as reference.

[b]Individuals who developed grade 2 alopecia.

[c]Individuals who did not developed any ADRs after chemotherapy.

[d]Paclitaxel and docetaxel administrated samples.
