# Supplementary material for: A genome-wide association study of chemotherapy-induced alopecia in breast cancer patients
Source: Breast Cancer Res. 2013 Sep 11;15(5):R81. doi: 10.1186/bcr3475 (PMC3978764; doi:10.1186/bcr3475)
Supplement: Additional file 8: Table S5 — Weighted genomic risk score of each genome-wide association study for chemotherapy-induced alopecia. [file bcr3475-S8.pdf]

Supplementary Table 5 wGRS of each GWAS for chemotherapy-induced alopecia

All (8 SNPs)

Cat	Score	ADR[a]	Non-ADR[b]	%_ADR	%_Non-ADR	OR[c]	95%_CI	P-value	Sensitivity	Specificity
1	<5.54	17	173	0.06	0.20			REF		
2	5.54-6.84	46	251	0.15	0.29	**1.87**	1.04-3.36	3.83E-02	0.73	0.41
3	6.84-8.22	186	426	0.61	0.49	**4.44**	2.62-7.53	3.44E-09	0.92	0.29
4	>8.22	54	28	0.18	0.03	**19.6**	9.99-38.6	1.44E-21	0.76	0.86
		303	878	1.00	1.00					

CEF (4 SNPs)

Cat	Score	ADR	Non-ADR	%_ADR	%_Non-ADR	OR	95%_CI	P-value	Sensitivity	Specificity
1	<1.94	4	47	0.03	0.44			REF		
2	1.94-3.54	40	33	0.35	0.31	**14.2**	4.65-43.7	3.51E-08	0.91	0.59
3	3.54-5.15	28	20	0.24	0.19	**16.5**	5.10-53.1	7.47E-08	0.88	0.70
4	>5.15	44	6	0.38	0.06	**86.2**	22.8-326	2.46E-17	0.92	0.89
		116	106	1.00	1.00					

CAF (8 SNPs)

Cat	Score	ADR	Non-ADR	%_ADR	%_Non-ADR	OR	95%_CI	P-value	Sensitivity	Specificity
1	<15.3	0	16	0.00	0.59			REF		
2	15.3-21.2	9	10	0.14	0.37	**29.9***	1.57-569	1.98E-03	1.00	0.62
3	21.2-27.1	42	1	0.66	0.04	**935***	36.2-24100	3.35E-14	1.00	0.94
4	>27.1	13	0	0.20	0.00	**891***	16.6-47900	3.77E-09	1.00	1.00
		64	27	1.00	1.00					

Anti-microtubules (6 SNPs)

Cat	Score	ADR	Non-ADR	%_ADR	%_Non-ADR	OR	95%_CI	P-value	Sensitivity	Specificity
1	<5.51	1	33	0.01	0.31			REF		
2	5.51-7.59	26	52	0.22	0.50	**16.5**	2.14-128	5.13E-04	0.96	0.39
3	7.59-9.68	65	19	0.55	0.18	**113**	14.5-881	1.06E-14	0.99	0.64

4	>9.68	26	1	0.22	0.01	**858**	51.2-14400	5.82E-15	0.96	0.97
		118	105	1.00	1.00					

Paclitaxel (7 SNPs)

Cat	Score	ADR	Non-ADR	%_ADR	%_Non-ADR	OR	95%_CI	P-value	Sensitivity	Specificity
1	>3.02	0	25	0.00	0.41			REF		
2	3.02-7.48	6	25	0.11	0.41	**13.0***	0.70-243	6.28E-02	1.00	0.50
3	7.48-11.9	35	11	0.61	0.18	**157***	8.87-2800	7.87E-11	1.00	0.69
4	>11.9	16	0	0.28	0.00	**1680***	31.8-89100	9.70E-12	1.00	1.00
		57	61	1.00	1.00					

Docetaxel (4 SNPs)

Cat	Score	ADR	Non-ADR	%_ADR	%_Non-ADR	OR	95%_CI	P-value	Sensitivity	Specificity
1	<2.26	1	24	0.02	0.57			REF		
2	2.26-4.78	9	14	0.15	0.33	**15.4**	1.76-135	3.80E-03	0.90	0.63
3	4.78-7.29	39	4	0.63	0.10	**234**	24.7-2220	3.11E-13	0.98	0.86
4	>7.29	13	0	0.21	0.00	**441***	16.8-11600	6.46E-10	0.93	1.00
		62	42	1.00	1.00					

* OR calculated after Haldane's correction: adding 0.5 to all the cells of a contingency table if any of the cell expectations would cause a division by zero error.

Cat, category; CEF, cyclophosphamide+epirubicin+/-5FU; CAF, cyclophosphamide+doxorubicin+/-5FU; ADR, adverse drug reaction; OR, odds ratio; CI, confidence interval; REF, reference.

[a]Individuals who developed grade 2 alopecia.

[b]Individuals who did not developed any ADRs after chemotherapy.

[c]ORs and Cis are calculated using category (group) 1 as reference.
