# Supplementary material for: A genome-wide association study of chemotherapy-induced alopecia in breast cancer patients
Source: Breast Cancer Res. 2013 Sep 11;15(5):R81. doi: 10.1186/bcr3475 (PMC3978764; doi:10.1186/bcr3475)
Supplement: Additional file 9: Table S6 — Weighted genomic risk score results of all, CAF- and CEF-induced alopecia. [file bcr3475-S9.pdf]

Supplementary Table 6 wGRS results of all, CAF and CEF-induced alopecia

ALL (8 SNPs)

Cat	Score	G2[a]	G1[b]	G0[c]	%_G2	%_G1	%_G0	G2 vs G0			G1 vs G0		
								OR[d*]	95%_CI	P-value	OR[d*]	95%_CI	P-value
1	<5.45	17	28	167	0.08	0.13	0.79		Ref			Ref	
2	5.45-6.83	46	52	242	0.14	0.15	0.71	1.87	1.04-3.37	3.79E-02	1.28	0.78-2.11	3.83E-01
3	6.83-8.21	186	66	417	0.28	0.10	0.62	4.38	2.58-7.43	1.11E-08	0.94	0.59-1.52	9.02E-01
4	>8.21	54	10	26	0.60	0.11	0.29	20.4	10.3-40.4	1.08E-21	2.29	1.00-5.27	5.34E-02
	**Total**	**303**	**156**	**852**									

ACF (8 SNPs)

Cat	Score	G2	G1	G0	%_G2	%_G1	%_G0	G2 vs G0			G1 vs G0		
								OR	95%_CI	P-value	OR	95%_CI	P-value
1	<15.6	0	3	18	0.00	0.14	0.86		Ref			Ref	
2	15.6-21.1	8	13	8	0.28	0.45	0.28	37	1.91-718	7.09E-04	9.75	1.27-3.99	3.56E-03
3	21.1-26.6	42	14	1	0.74	0.25	0.02	1048	40.8-26953	1.45E-14	84	7.86-897	3.04E-06
4	>26.6	14	3	0	0.82	0.18	0.00	1073	20.1-57414	2.12E-09	37	1.55-886	9.81E-03
	**Total**	**64**	**33**	**27**									

CEF (4 SNPs)

Cat	Score	G2	G1	G0	%_G2	%_G1	%_G0	G2 vs G0			G1 vs G0		
								OR	95%_CI	P-value	OR	95%_CI	P-value
1	<1.96	4	9	5	0.22	0.50	0.28		Ref			Ref	
2	1.96-3.54	40	14	31	0.47	0.16	0.36	1.61	0.40-6.51	7.24E-01	0.251	0.07-0.89	3.33E-02
3	3.54-5.12	28	10	20	0.48	0.17	0.34	1.75	0.42-7.35	4.85E-01	0.278	0.07-1.05	1.01E-01
4	>5.12	44	6	4	0.81	0.11	0.07	13.8	2.60-72.8	2.89E-03	0.833	0.16-4.44	1.00E+00
	**Total**	**116**	**39**	**60**									

* OR calculated after Haldane's correction: adding 0.5 to all the cells of a contingency table if any of the cell expectations would cause a division by zero error.

Cat, category; OR, odds ratio; CI, confidence interval; REF, reference.

[a]Individuals who developed grade 2 alopecia.

[b]Individuals who developed grade 1 alopecia.

[c]Individuals who did not developed any ADRs after chemotherapy.

[d]ORs and CIs are calculated using category (group) 1 as reference.
